# Artificial Intelligence in the Diagnosis of Colorectal Cancer: A Literature Review

**DOI:** 10.3390/diagnostics14050528

**Published:** 2024-03-01

**Authors:** Petar Uchikov, Usman Khalid, Krasimir Kraev, Bozhidar Hristov, Maria Kraeva, Tihomir Tenchev, Dzhevdet Chakarov, Milena Sandeva, Snezhanka Dragusheva, Daniela Taneva, Atanas Batashki

**Affiliations:** 1 Department of Special Surgery, Faculty of Medicine, Medical University of Plovdiv, 4002 Plovdiv, Bulgaria; puchikov@yahoo.com (P.U.); tihomir.tenchev88@gmail.com (T.T.); a_batashki@yahoo.com (A.B.); 2 Faculty of Medicine, Medical University of Plovdiv, 4002 Plovdiv, Bulgaria; usmankhalid957@gmail.com; 3 Department of Propaedeutics of Internal Diseases “Prof. Dr. Anton Mitov”, Faculty of Medicine, Medical University of Plovdiv, 4002 Plovdiv, Bulgaria; 4 Section “Gastroenterology”, Second Department of Internal Diseases, Medical Faculty, Medical University of Plovdiv, 4002 Plovdiv, Bulgaria; hristov.bozhidar@abv.bg; 5 Department of Otorhinolaryngology, Medical Faculty, Medical University of Plovdiv, 4002 Plovdiv, Bulgaria; kraevamaria93@gmail.com; 6 Department of Propaedeutics of Surgical Diseases, Section of General Surgery, Faculty of Medicine, Medical University of Plovdiv, 4002 Plovdiv, Bulgaria; dchakarov@mail.bg; 7 Department of Midwifery, Faculty of Public Health, Medical University of Plovdiv, 4000 Plovdiv, Bulgaria; sandewa@abv.bg; 8 Department of Nursing Care, Faculty of Public Health, Medical University of Plovdiv, 4000 Plovdiv, Bulgaria; sdragusheva68@gmail.com (S.D.); taneva.daniela@abv.bg (D.T.)

**Keywords:** artificial intelligence, colorectal cancer, diagnosis, autonomous learning, advanced neural software

## Abstract

Background: The aim of this review is to explore the role of artificial intelligence in the diagnosis of colorectal cancer, how it impacts CRC morbidity and mortality, and why its role in clinical medicine is limited. Methods: A targeted, non-systematic review of the published literature relating to colorectal cancer diagnosis was performed with PubMed databases that were scouted to help provide a more defined understanding of the recent advances regarding artificial intelligence and their impact on colorectal-related morbidity and mortality. Articles were included if deemed relevant and including information associated with the keywords. Results: The advancements in artificial intelligence have been significant in facilitating an earlier diagnosis of CRC. In this review, we focused on evaluating genomic biomarkers, the integration of instruments with artificial intelligence, MR and hyperspectral imaging, and the architecture of neural networks. We found that these neural networks seem practical and yield positive results in initial testing. Furthermore, we explored the use of deep-learning-based majority voting methods, such as bag of words and PAHLI, in improving diagnostic accuracy in colorectal cancer detection. Alongside this, the autonomous and expansive learning ability of artificial intelligence, coupled with its ability to extract increasingly complex features from images or videos without human reliance, highlight its impact in the diagnostic sector. Despite this, as most of the research involves a small sample of patients, a diversification of patient data is needed to enhance cohort stratification for a more sensitive and specific neural model. We also examined the successful application of artificial intelligence in predicting microsatellite instability, showcasing its potential in stratifying patients for targeted therapies. Conclusions: Since its commencement in colorectal cancer, artificial intelligence has revealed a multitude of functionalities and augmentations in the diagnostic sector of CRC. Given its early implementation, its clinical application remains a fair way away, but with steady research dedicated to improving neural architecture and expanding its applicational range, there is hope that these advanced neural software could directly impact the early diagnosis of CRC. The true promise of artificial intelligence, extending beyond the medical sector, lies in its potential to significantly influence the future landscape of CRC’s morbidity and mortality.

## 1. Introduction

Colorectal cancer (CRC) is the third most common cancer, and its malignancy is the second deadliest for males and females combined [1]. Its rising incidence in western countries can be attributed to obesity, which increases the levels of visceral adipose tissue, promoting the development of proinflammatory cytokines and inducing an inflammatory effect on the colon. Poor nutrition feeds the risk of development by up to 70%, with red meats stimulating the formation of N-nitroso carcinogenic compounds. Recreational smoking and the consumption of alcohol stimulates metabolite production, with nicotine and acetaldehyde contributing as risk factors. The aetiology of CRC is thought to involve a mutation in the adenomatous polyposis coli suppressor gene, triggering the formation of non-malignant polyps, which can give rise to malignant states up to 10 years after polyp formation. A hereditary predisposition involving the familial adenomatous polyposis undergoes point mutations, stimulating multiple potentially malignant polyps to form in the colon. Chromosomal instability, microsatellite instability, and the CpG island methylator phenotype are major molecular pathways of colorectal cancer, which are associated with the loss of heterozygosity, loss of DNA repair mechanism, and gene silencing [2]. 

The most impactful diagnostic tool for colorectal cancer is the endoscope. This allows for tumour localisation and histological examination through tissue biopsy. With a specificity and sensitivity of above 90%, it has proven to be a vital tool to reduce morbidity and mortality. Despite this, endoscopic analysis has its drawbacks. The discomfort of an endoscopy has a certain fear factor, which discourages patients from undergoing the exam; moreover, the risk of intestinal perforation and bleeding makes patients unwilling to participate [3]. In a study investigating the benefits of surveillance endoscopies in patients with CRC, the conclusion indicated that surveillance did not improve survival for patients for local or regional CRC [4]. Imaging tests such as CT, abdominal ultrasonography, roentgenography of the thorax, and NMR are only supportive in advanced focal lesions, highlighting their ineffective role in the early diagnosis of CRC [3]. Therefore, it is imperative to enhance diagnostic tools to facilitate an early diagnosis and minimise the risk of mortality.

The emergence of artificial intelligence in medical diagnosis has a pivotal role in early pathology discovery, which provides a more targeted and effective approach to cancer therapy. Artificial intelligence is a novel term which encompasses a spectrum of methodologies and neural networks that enhance existing tools or serve as a new outlook in the prevention, diagnosis, and treatment of disease. The autonomous and expansive learning ability of artificial intelligence, coupled with its ability to extract increasingly complex features from images or videos without human reliance, highlights its impact in the diagnostic sector. Its speed and specificity are enhanced when compared to human intervention [5]. 

This paper aims to describe how artificial intelligence is changing the way colorectal cancer is diagnosed, with a particular emphasis on its impact on future rates of illness and death related to colorectal cancer. We want to thoroughly examine AI’s impact by exploring its complexities, including the problems, potentials, and necessity of various datasets to improve the accuracy and effectiveness of neural models.

We are examining the existing use of AI in diagnosing CRC to establish a thorough grasp of its strengths and weaknesses. We aim to explain how current research endeavours focused on improving brain structures and broadening the range of AI uses have the potential to significantly influence early CRC detection. As we begin this study, we foresee an advanced diagnostic environment where AI, along with human expertise, significantly influences the reduction in CRC morbidity and mortality, leading to better patient outcomes and improvements in the quality of healthcare.

## 2. Method

A targeted, non-systematic review of the published literature relating to colorectal cancer diagnosis was performed, with PubMed databases being scouted to help provide a more defined understanding of the recent advances regarding artificial intelligence and their impact on colorectal-related morbidity and mortality. Different combinations of keywords and phrases were used to narrow down and find relevant source material. Keywords included but were not limited to colorectal cancer diagnosis, artificial intelligence, neural learning, machine learning, deep learning, and automation. All material published before 29th January 2024 was eligible for inclusion in this review. Articles that were not published in English were not included in this literature review. Articles were included if deemed relevant and including information associated with the keywords.

## 3. Discussion

### 3.1. Molecular Biomarkers

Precision oncology in colorectal cancer requires an expansive analysis of the genetic biomarkers involved in pathology development. Targeted biomarker research is a topic of interest for researchers, as they develop intelligent learning programs to improve pattern recognition and data set organisation. The expansion of machine learning has provided a breakthrough in the predication of molecular instabilities. A diagnostic study using a deep learning model, MSINet, was developed using 100 H&E-stained, whole slide images from patients who underwent colorectal cancer resection. The learning tool outperformed experienced gastrointestinal pathologists in predicting microsatellite instabilities, with the model reaching a negative predictive value of 93.7% [6]. A similar pathomic-based model was developed through a multiple-instance-learning, deep learning model to create an ensembled patch likelihood aggregation (EPLA) based on patch-level prediction and whole slide images. When distinguishing the EPLA model with state-of-the-art deep learning, it consistently outperformed its comparative model, with an AUC of 0.8848, when analysing whole slide images. The reliability was further verified by carrying out the experiment into two individual cohorts, which highlighted its variability when presented with alternating data sets of patients from different continental backgrounds. To directly compare the method with the DL-based MV in the same test set, a deep-learning-based, majority-voting method in the TCGA-COAD cohort was developed and achieved an AUC of 0.8457, consistent with the result in Kather’s study [7]. A further comparison of the specificity and sensitivity of the two components of the EPLA with those of deep-learning-based majority voting found that the bag of words technique achieved a higher specificity (89.5% vs. 75.2%) and PAHLI sensitivity (86.4% vs. 81.8%). The ensembled EPLA classifier combined the advantage of its two components, obtaining superior specificity and sensitivity compared to deep-learning-based majority voting. All codes were implemented in Python and run on a workstation with Nvidia GPUs (P40). The average time to complete a single patient test was 0.5118 s on a P40 workstation and 20.9291 s on a regular CPU machine (16 GB, 3.00 GHz, i5-9500). The utilisation of patient data sets from The Cancer Genome Atlas eliminated the need for immunohistochemical and genetic testing [8]. To establish a breakthrough in the use of conventional neural networks and advance the sensitivity and data efficiency of deep learning networks, the integration of a transformer-based pipeline for end-to-end biomarker prediction was created through a combination of a pre-trained transformer encoder and a transformer network for patch aggregation. The results from the transformer-based approach yielded a higher peak performance compared to a classic patch approach when assessed in both large and small cohort groups. Furthermore, a negative predictive value of 0.99 was obtained for microsatellite instability prediction [9]. Alongside MSIs, polygenic state predictions are also vital in CRC diagnosis. Bilal et al. used ResNet34 and ResNet 18 to report algorithms that predicted multi-gene expression status alongside BRAF, TP53, CpG island methylation, and chromosome status. The devices were trained on balanced datasets of tumour and non-tumour tiles, which served as input to iterative draw and rank sampling. Informative tiles were obtained through tissue segmentation and tile extraction. By using the same patient cohort and splitting the dataset into training and testing groups, they compared their prediction of microsatellite instability with that of Kather and colleagues [7]. The iterative draw and rank sampling method outperformed the method used by Kather and colleagues, with a mean AUROC of 0.90 versus 0.77. The results yielded AUCs over 0.9 in internal datasets, suggesting the viability of ResNet in pattern recognition for genomic biomarkers. The proposed algorithm for predicting clinically valuable mutations and molecular pathways, such as MSIs, in colorectal cancer could be used to stratify patients for targeted therapies with quicker turnaround times compared to sequencing-based or immunohistochemistry-related approaches [10]. Semi-supervised learning systems use both labelled and unlabelled data and have achieved excellent results in nature image processing; furthermore, they outperform supervised learning programs, indicating their diagnostic use for colorectal cancer [11]. Afshar et al. investigated the application of an artificial neural network in miRNA biomarker selection for the precise diagnosis of CRC. A MATLAB multilayer perceptron ANN model with three layers was developed. The input layer comprised four neurons, representing four selected miRNAs. The hidden layer had seven neurons, while the output layer had a single neuron, indicating either a healthy control (0) or cancer (1). To optimize training parameters, various miRNA sets were assessed. Initially, the model was trained using the miRNA with the highest score. Then, additional miRNAs were incrementally added based on their scores, and the model’s performance was evaluated after each addition. This iterative process continued until the optimal performance was attained. MiRNAs retrieved from the Gene Expression Omnibus were evaluated due to their correlation with the signalling pathway of CRC. The outcomes of the simulation revealed the proficient capability of the engineered Artificial Neural Network (ANN) model in precisely discerning between cancerous and non-cancerous sample data. Moreover, the assessment of the ANN model yielded noteworthy observations: the Area Under the ROC curve (AUC), alongside the regression coefficient, denoting the correlation between the ANN’s output and the anticipated output, was one. Additionally, the confusion matrix of the ANN model conspicuously demonstrated the accurate classification of non-cancerous patients as normal and cancerous patients as having cancer [12]. The detection of the BRAF V600E mutation is viewed as a rapid, low-cost, and sensitive method, which utilises near-infrared (NIR) spectroscopy enhanced by counter-propagation artificial neural networks (CP-ANN). A total of 104 paraffin-embedded CRC samples were used to calibrate the algorithm. The NIR detection incorporates molecular differences to discern between BRAF V600E and wild-type mutations based on their intrinsic differences, giving the instrument a high level of sensitivity. The CP-ANN model demonstrated a diagnostic sensitivity of 100.0%, diagnostic specificity of 87.5% and a diagnostic accuracy of 93.8%. When compared to the time-consuming PCR and gene sequencing, the NIR detection is sensitive, does not require sample preparation, and is inherently rapid. This standout performance reinforces the power of AI integration with instrumental analysis in the diagnosis of CRC [13]. In a separate study, artificial intelligence was used in a database to visually represent the intricate connections among the various factors associated with DNA methylation in CRC. The ANN models were able to detect connections between the studied variables, such as MLH1 hypermethylation and right colon disposition, and the novel factors associated with the variables. Their ability to amalgamate environmental, epigenetic, genetic, and disease-related information could provide an opportunity to link geno-environmental factors to CRC. Neural learning assists in stratifying large cohorts of CRC samples into identifiable molecular subtypes for a more complete understanding of the environmental factors linked to each of them [14]. 

### 3.2. Instrumental Integration of Artificial Intelligence

With the colonoscopy being the lead in the diagnostics of colorectal cancer, methods must be devised to incorporate it with artificial intelligence. GI-Genius is based on a convolutional neural network that pools data from a large cohort of patients with histologically confirmed polyps. When GI-Genius is used during the insertion and withdrawal phases, it identified any suspicious lesions or polyps through computer-aided diagnosis endoscopy. The AI tool demonstrated a reduced risk of miss rate by around 50% compared to a regular endoscopy, primarily associated with its increased detection rate in polyps < 10 mm. The reduction in false negative rate, compared to a regular endoscopy (6.8% vs. 29.6%), highlights its identifying role in adenoma detection [15]. Incidentally, the adenoma miss rate was investigated in a multi-centre randomized tandem colonoscopy in the United States, with the results reinforcing the use of deep learning [16]. The complexities of deep learning architecture were explored by Wang et al. when creating a deep neural network based on SegNet architecture. With preliminary studies highlighting promising results, specificity reached 95.92% and per-image sensitivity reached 94.39%. The study demonstrated a significant rise in PDR, ADR, and average polyps and adenomas per colonoscopy within the CADe group compared to the control group. However, the elevated adenoma detection primarily stemmed from the detection of more diminutive adenomas. The CADe system predominantly identified smaller diminutive adenomas, aligning with the conventional understanding that smaller polyps are more prone to being visually overlooked. Despite diminutive adenomas posing lower malignancy risks than larger ones, the overall increase in adenoma detection rate through CADe technology could potentially reduce the risk of interval CRC. The increase in adenoma detection rate relative to a regular endoscopic exam highlights the reliability of the tool [17]. To access the true benefits of artificial intelligence, Song et al. utilised 12,480 image patches of 624 polyps to train the computer-aided diagnostic system. To create a more sensitive computer model, the intelligent system was trained to classify the pictures into three histological types: serrated polyps, benign adenomas, and deep submucosal cancer. As a result, the Kappa values shot up for trainees using the artificial intelligence to aid in polyp detection, from 0.368 to 0.655, with the overall diagnostic accuracy increasing from 63.8–71.9% to 82.7–84.2%. The average inference time for histological assessment was 0.02 s with ResNet-50 and 0.04 s with DenseNet-201. The rapid interference times reinforce its ability to be used in a clinical scenario where diagnosis must be rapid and early diagnosis has a strong impact on patient health [18]. Despite the success of the computer-aided diagnostic system, Yao et al. [19] set out to further increase the adenoma detection rates by finetuning the system through combining it with computer-aided quality-improvement systems, which further sharpened its detection rates by up to 30.60% compared to the 21.27% enhancement achieved using a computer-aided diagnostic system and 24.54% obtained using a computer-aided quality-improvement system. In 2020, Gong et al. [20] employed a neural network system involving ENDOANGEL, which advanced adenoma detection rates by up to 16% compared to control groups, who achieved an enhancement 8% [21]. In a pilot study conducted by Misawa and colleagues, a dataset composed of 73 colonoscopy videos was utilized, featuring 155 polyps. Within the dataset, flat lesions comprised around 64.5% of the cases, and each frame containing a polyp was retrospectively annotated and analysed by two specialised endoscopists, which served as the reference for polyp presence. The dataset was split into 155 polyp-positive and 391 polyp-negative short videos, which were randomly assigned for training and assessing the Convolutional Neural Network. The algorithm outperformed four novice endoscopists (with less than one year of colonoscopy experience) and demonstrated a comparable performance to two experts. The DNN-CAD provided diagnoses in 0.45 s, significantly faster than both expert endoscopists (1.54 s) and non-experts (1.77 s). The algorithm outperformed four novice endoscopists (with less than one year of colonoscopy experience) and demonstrated a comparable performance to two experts. The DNN-CAD provided diagnoses in 0.45 s, significantly faster than both expert endoscopists (1.54 s) and non-experts (1.77 s). A threshold of 15% probability was established for polyp detection based on a receiver operating characteristic analysis. The convolutional neural network (CNN) system reached a sensitivity of 90.0%, a specificity of 63.3%, and an accuracy of 76.5% through image-frame-based analysis of a test set comprising 135 short videos [22]. 

### 3.3. Neural Enhancement of MRIs

In cancer diagnosis, a precise interpretation of the findings is necessary for staging and therapeutic management. The segmentation of rectal cancer was enhanced through an artificial intelligence software, which estimated the area of the tumour, rectum, and mesorectum. The network comprises encoder and decoder sections with skip connections. Each convolutional block includes a 3D convolution layer, batch normalization, and ReLU activation. Deconvolution blocks use transposed convolution operators. Skip connections involve 1 × 1 × 1 convolutions, batch normalization, and ReLU. Input is a 3D MR image and output matches input dimensions with three channels each for mesorectum, rectum, and tumour probabilities. Segmentation is achieved by thresholding at 0.5. The algorithm calculates the T stage, following the binary segmentation results. As T2 MR images are more commonly used by radiologists in the preoperative diagnosis of colorectal cancer, the study was performed in 3D through T2-weighted MR images using a U-Net deep neural network. Analysis was performed on the images of 201 patients who underwent pre-operative MRI scans for data training. The algorithm evaluated segmentation accuracy and staging accuracy. It successfully estimated the area of the tumour, rectum, and mesorectum. In diagnostics, differentiation between stage T2 and T3 is imperative from a therapeutic standpoint. Therefore, when investigating the diagnostic differentiation capacity of the AI, the T-stage sensitivity, specificity, and accuracy at 0.773, 0.768 and 0.771, respectively, demonstrated an enhanced T stage performance when compared to a baseline model, which achieved scores of 0.765, 0.756, and 0.761, respectively. The objective analysis of the AI algorithm demonstrates its aid risk stratification and tailoring of individual treatments due to its ability to individualise T stages [23]. Fang et al. opted to use dark-lumen magnetic resonance imaging based on an AI algorithm to explore the diagnostic impact in colon cancer. The study involved 98 patients with ulcerative-type colon cancer, with the study aiming to establish the diagnostic efficacy and value of the dark-lumen-based MRI and its neural integration. The apparent diffusion coefficient values of patients in the algorithm group had comparatively higher values (1.55 ± 0.31 mm^2^/s) compared to the control group (0.92 ± 0.14 mm^2^/s), which indicated a statistically significant difference, highlighting the AI algorithms’ efficacy. ME and Er indicators displayed diagnostic value by evaluating the lesion signal display effect of the two groups of MR colonography with the algorithm that exceeded both values when compared to the control group. Furthermore, the mean value for the algorithm (10.42) was almost double that of the control (5.27), reinforcing its accuracy in the judgement of invasion depth [24]. Faster, region-based, convolutional neural networks divided the MRIs into three planes (coronal, sagittal, and horizontal), demonstrating two components, tumour segmentation and stage detection, for each. The faster CNN is composed of region proposal networks (RPN), convolutional layers, a region of interest, pooling, and classification. The RPN was utilised to train the network, with finetuning performed by the convolutional layers. A combination of advanced autonomic features provides the AI with a stable platform for image recognition and judgement. After 50 epochs of learning, the AI code reached 100% accuracy in automatic image plane identification, which suggests that the algorithm could correctly distinguish the projection plane of the CRC data collected from the MRI. AUC analysis fortified the platform’s excellent performance for every plane in every stage [25]. The fusion of AI and medical imaging yielded a synergistic effect. Within the realm of computational science, this innovative AI integration extends the scope and diversifies the application landscape of artificial intelligence. It empowers AI to bolster productivity and foster advancement within a novel domain, bolstering the influence of computational technology.

### 3.4. Hyperspectral Imaging

Hyperspectral imaging (HSI) is a non-invasive imaging technique that uses a broadband light source to measure optical tissue properties across different electromagnetic bands. Due to their complex nature, algorithms such as convolutional neural networks, support vector machines (SVM), and multilayer perceptron (MLP) learn patterns in a hierarchal way, which takes advantage of a 3D data structure and helps to discriminate between CRCs. Collins et al. explored the imaging technique on a dataset of twelve patients, with three parameters evaluated to determine its application in a practical scenario. The ROC-AUC of the 3DCNN exceeded the SVM and MLP by an area of 0.04. With a dice score of 0.41, the 3DCNN had the best scoring relative to the other models tested. The performance of both the RBF and MLP models showed an improvement when trained on the combined dataset compared to training solely on the esophagogastric dataset. This improvement was consistent regardless of whether a patient-generic or a patient-specific decision threshold was used. Notably, across all models, employing a patient-specific decision threshold resulted in a significantly better performance compared to a patient-generic threshold. The 3DCNN performed significantly better relative to the other models without training on the combined dataset. When trained on the combined dataset, improved results were obtained by the RBF and MLP models for the esophagogastric dataset. These optimistic results suggest that a spectral-based CAD system using an interactive decision threshold has the potential to be valuable. Moreover, with experiments combining colorectal and esophagogastric datasets, the results showed a drastic improvement in the SVM and MLP models [26]. Research into combined Fourier transform infrared (FTIR) hyperspectral imaging has become a point of interest due to its role in representing different pathological states in biopsy slides. Furthermore, the training procedure spanned 500 epochs with a batch size of 5120, considering an overall count of 67,500 voxels. With a six-layer, fully connected, neural network architecture, the deep learning model, alongside the micro-FTIR-HSI, demonstrated spectacular pattern recognition capabilities, with a clear ability to distinguish between cancerous, healthy, and inflammatory tissue, allowing it to achieve up to 100% accuracy in most of the tested folds. When compared to other benchmark neural learning software, HSI outperformed the traditional neural networks with linear SVM, achieving the highest comparable accuracy of 96.48% [27]. In a separate four-layer perceptron neural network, Winkeln et al. investigated the ability of the algorithm to individualize tissue states into cancer, adenomatous margins around the central tumour, and healthy mucosa. Their classification resulted in an 86% sensitivity, with a specificity of 95% for cancer and adenomatous margins. Moreover, an AUC score of 97 demonstrates its sharp differentiation between adenoma and healthy mucosa. Specific spectral signatures developed by the tissue-light interactions mediate for tissue perfusion assessment and tissue differentiation. Physiological tissue parameters, including tissue oxygenation (StO2), near-infrared perfusion index (NIR PI), tissue water index (TWI), and organ haemoglobin index (OHI), were assessed with healthy mucosa, showing a lower TWI, OHI, and StO2 than cancer, whereas adenomas displayed a higher TWI, OHI, and StO2 than healthy mucosa. The interpretation of tissue physiology allows for a well-rounded evaluation of the cancer history, providing a more thorough diagnostic and therapeutic standpoint [28]. 

### 3.5. Deep Neural Network Architecture

Dulf et al. explored neural networks, with the aim of reducing the accuracy lost during the decisional process, which is associated with an increase in the number of modulatory factors. Five different types of architecture were tested and compared through typical classification problems. Sensitivity and F1 were the most weighted criteria in choosing decisive networks, with Inception-v3 being selected and outscoring the other algorithms (a sensitivity of 98.13% and an F1 score of 98.14%). Inception-v3 is based on the GoogleNet model, Inception-v1, and works to reduce the degree of “strangulation” of the network caused by the convolutional layers. This allows for a mitigation in the number of parameters by a further 33%. With 42 layers, its computation effort is 2.5 times greater, but its efficiency surpasses that of the VGGNet model. For the identification of malignant areas, the F1 score and Jaccard index were the preferred indices due to their varying classification metrics. The model trained on the “Kvasir-Seg” set with augmentation was selected for use in the application, obtaining an F1 score of 54.01% and a Jaccard index of 75.18% [29]. As endoscopic techniques were highly operator-dependent, the results of the instrumental analysis varied and could highlight human error. To overcome this, Choi et al. applied deep learning to computer-aided diagnostics trained with 3000 images, dividing the results into four categories: normal, low-grade dysplasia, high-grade dysplasia, and adenocarcinoma. They used Inception-v3, ResNet-50, and DenseNet-161 as baselines. However, the researchers altered the models to improve their balancing and transfer learning. To determine the mark and accuracy of the models, they were compared to endoscopists of varying experience, with the neural networks all outperforming the expert group. Furthermore, the model visualization results showed regions of interest to explain the classification decision of the pathology, allowing for an analysis of the models’ reasoning [30]. Deep neural networks with fragment length and methylation signatures were implemented for blood-based assays in the development of SPOT-MAS, which provides a high accuracy for early CRC detection. This is vital due to the aggressive nature of the cancer. The model attained a 0.989 AUC and a sensitivity of 96.8% whilst maintaining a 97% specificity in detecting CRC. At the same time, the model’s external validation demonstrated similar effectiveness, yielding a 0.96 area under the curve. Its ability to differentiate blood samples from healthy patients and patients with cancer is imperative when considering a personalised therapeutic approach to reduce mortality [31]. To truly grasp the fundamental importance of deep neural networks, we need to evaluate their use in other areas outside of the CRC to serve as a cross-reference for its viability. Fekri-Ershad et al. developed a tuned three-layer perceptron fed with trained deep CNNs for cervical cancer diagnosis. The utilization of ResNet34 and ResNet50 with VGG-19 yielded promising results, with the CNN being trained on images using the Adam optimizer. Machine learning approaches typically involve two key stages: feature extraction and classification. In these methods, features are initially derived from Pap smear images, followed by a training phase. During training, the system learns to discern patterns from the extracted features. Subsequently, in the classification phase, the trained system assigns labels to test images based on these learned patterns. In deep learning methods, feature extraction occurs within the learning phase itself, seamlessly integrating the process into the network architecture. Unlike traditional approaches, deep networks utilize various layers, such as fully connected or SoftMax layers, in the final stages to classify test images based on the extracted features. The proposed method was evaluated using the Herlev benchmark database and provided a 99.23 percent accuracy for the two-classes case and 97.65 percent accuracy for the seven-classes case. The results demonstrate a higher accuracy compared with the baseline networks and many existing methods [32]. Fekri-Ershad et al. further introduced an efficient method for diagnosing cervical cancer in pap smear images by employing a multistage algorithm: initially, a basic thresholding technique eliminates the cytoplasm part, encompassing the nucleus, from the intracellular fluid in cervix cells. As a result, the study reveals significant changes in the nuclear texture and cytoplasm of cancer cells compared to healthy cells, prompting the introduction of a novel local texture descriptor named modified uniform local ternary patterns (MULTP) for extracting discriminative local texture features. Finally, a multi-layer neural network was embedded for classification and enhanced using a genetic algorithm to optimize the neural network’s architecture, specifically the number of hidden nodes and layers, thereby improving overall performance. The promising extra-CRC involvement of deep neural networks further proved its efficacy in an expanding the AI-based diagnostic future in medicine [33].

Table 1 was created to highlight individual AI models to distinguish between the studies and their independent conclusions.

### 3.6. The Lack of Clinical Practicality in AI

With the dramatic and unprecedented rise of artificial intelligence, its use has been hindered in practical medicine due a lack of trials and testing, which prevent its implementation into modern medicine. When predicting microsatellite instability, downsampling from 40 times images to 20 times images restricted the inclusion of high-resolution information such as image texture and boundary information. Furthermore, with the model training being limited to a small sample group of patients, there could be a lack of diversity in patient information across a multitude of socio-economic and geographic backgrounds, necessitating an expansion in the training data to improve the generalizability and accuracy of the model [6]. Despite the advancements in MSI at the genomic level, the transcriptomic levels remain understudied. Also, as deep learning models are a recent innovation within the medical landscape, early adopters have criticised the algorithms for their poor interpretability, with only a few models being trusted and verified by clinicians in clinical practice [8]. Further testing from separate studies indicated that when the training data and the test data were not from the same source, there was a discrepancy in the results, yielding poorer results for the artificial intelligence models [11]. The advancement of precision oncology has been blockaded by its costly and complex process, alongside the intricate instrumentation and expertise that are required. Deep learning is limited by its controversial performance and it is uncertain whether it is applicable to a large population. It also cannot be generalised to any patient population and is not permitted to be used on biopsy materiel [9]. Confidence heatmaps show that both positively and negatively labelled tiles with varying confidence could be linked to the difficulties associated with the immunogenic response of microsatellite instability, like the histomorphology of microsatellite instability. This suggests that a more complex method of aggregation is required to classify molecular characteristics, beyond what is currently available [10]. There is an increased necessity that ANNs can achieve crosstalk amongst epigenetic, genetic, and environmental factors. This would allow for the differentiation of CRCs based on their genetic and epigenetic signatures, which would pave the way for the discovery of the most promising biomarkers, allowing for an early CRC diagnosis based on the subtype. This would serve as a perquisite for stratifying large cohorts of CRC samples into molecular-based divisions [14].

Some studies were not powered to detect differences in ADRs. Tandem colonoscopy provides us with important information regarding CAD performance but has a limited role in a general clinical setting. Also, given that endoscopists were not blinded during the study, it could be assumed that the performance of the endoscopist was based on its being observed, which would further affect the CAD’s recorded functionality. Moreover, given that some studies used only expert colonoscopists as their baseline comparison to the CAD, it is unclear how CAD-assisted colonoscopies will affect the endoscopy performance depending on who is managing the instrument and AI. This presents an overly complex situation where the user may hinder the true abilities of the neural networks rather than the intrinsic power of the model [16]. Though optical studies remain a promising area of research in CRC diagnosis, biopsy is still seen as the gold standard. The AI is also limited by how much surface microstructures can reflect the histo-morphological features of a lesion; if the program does not pick up a lesion, it completely rules out its diagnosis unless there is human intervention, which would ultimately take more time and would essentially remove the need for the tool in the first place. Adding onto the previous point, AI has a certain visual field and will act within these parameters. This excludes any polyp lying outside these parameters, as it will not be detected by the CAD system, creating a range problem for a tool as its effectivity is classified relative to the area it covers. For an automatic polyp detection system to be considered ‘real-world’, it must demonstrate rapid analysis times with no delay to the endoscopist, something which is not within the scope of current AI software. Another constraint is the absence of external legitimacy. The baseline adenoma and polyp detection rates in this study were not as high as those reported in Western countries. This could be due to a variety of variables, including genetic, nutritional, lifestyle, and habitus differences between Chinese and Western populations, as well as disparities in the morbidity of colon polyps/adenomas. As a result, the study’s findings may not be applicable to places in the world with a higher baseline ADR [17].

Although AI requires variables and data to make its decisions, specifics regarding patient data are not available for the algorithm. For example, in the study looking at T stage diagnosis, there were no patients who could not undergo surgery, and the study did not include patients who had a stage that was too advanced to receive surgery. The absence of such information in the algorithms’ learning process could evolve into inaccurate MRI assessments for similar patients. Also, as the depth of invasion and the pathological stage are different for different tumours at varying distances from the anus, this could impact the image assessment of the AI [25]. 

## 4. Conclusions

Since its inception in colorectal cancer, artificial intelligence has emerged as a transformative force, introducing a great number of functionalities and enhancements within the diagnostic approach to CRC. The expansive capabilities of AI, spanning from intricate genomic analysis to seamless collaboration with instruments like the endoscope, underscore the practicality and affirmative outcomes observed in the initial testing.

While the current body of research predominantly involves a limited sample size, it is imperative to underscore the necessity to diversify patient data. This diversification is not merely a procedural requirement but a critical step toward refining cohort stratification, which is essential for developing neural models that exhibit heightened sensitivity and specificity.

Although it is currently in the early stages of implementation, the clinical application of AI in CRC diagnosis appears to be on the horizon. The trajectory, however, demands steadfast dedication to ongoing research aimed at refining neural architecture and broadening the scope of applications. The anticipation is that these advanced neural solutions, through consistent improvements and expanded utility, will play a direct and impactful role in the early diagnosis of CRC.

The true promise of artificial intelligence, extending beyond the medical sector, lies in its potential to significantly influence the future landscape of CRC morbidity and mortality. As AI seamlessly integrates into various domains, including healthcare, it not only promises enhanced diagnostic capabilities but also presents an opportunity to redefine the quality of life. By synergizing human expertise with the computational prowess of intelligent algorithms, we are poised not just to advance diagnostics but to make substantial strides in improving patient outcomes, ultimately shaping a future where CRC is detected earlier and treated more effectively, consequently reducing the overall burden of morbidity and mortality.

## Figures and Tables

**Table 1:** AI Models and Independent Conclusions in Highlighted Studies.

Study	Publication Year	Method	Data Participants	AI Model Used	Conclusion
Yamashita R et al. [6]	2021	The MSINet model was trained on 100 H&E-stained WSIs (50 MSS, 50 MSI) from patients at Stanford University Medical Centre and internally validated on 15 WSIs. Externally, it was validated on 484 WSIs from The Cancer Genome Atlas. Performance metrics included sensitivity, specificity, NPV, and AUROC, compared with five pathologists’ assessments of a subset of 40× magnification WSIs (20 MSS, 20 MSI).	15 internally validated patients and 484 externally validation patients.	MSINet	The deep learning model exceeded the performance of experienced gastrointestinal pathologists in predicting MSI on H&E-stained WSIs.
Cao R et al. [8]	2020	Establishing the pathomics model, EPLA, based on two consecutive stages: patch-level prediction and WSI-level prediction. The initial model was formed and validated in TCGA-COAD, then generalized in Asian-CRC through transfer learning. The pathological signatures generated from the model were inspected with genomic and transcriptomic profiles for model interpretation.	429 patients	MIL deep learning to create an EPLA	Effective MSI prediction from histopathological imaging, which is transferable to a new patient cohort.
Wagner SJ et al. [9]	2023	Developing a new transformer-based pipeline for end-to-end biomarker prediction from pathology slides by combining a pre-trained transformer encoder with a transformer network for patch aggregation.	>13,000 patients	Convolutional Neural Networks	A sensitivity of 0.99 and a negative predictive value > 0.99 were achieved for the prediction of MSIs on surgical resection specimens. Resection specimen-only training reached clinical-grade performance on endoscopic biopsy tissue, solving a long-standing diagnostic problem.
Bilal M et al. [10]	2021	Tumour tiles were processed by models trained using iterative draw and rank sampling to predict molecular labels such as high mutation density, microsatellite instability, chromosomal instability, CpG island methylator phenotype (CIMP)-high (vs. CIMP-low), BRAFmut, TP53mut, and KRASWT. The resulting scores identified top-ranked tiles, which were then analysed by model 3 (HoVer-Net) for cell nuclei segmentation and classification. Model performance was assessed using the area under the convex hull of the receiver-operating characteristic curve (AUROC) and compared with prior methods.	499 patients	ResNet18 and ResNet34	After large-scale validation, the proposed algorithm for predicting clinically important mutations and molecular pathways, such as MSI, in colorectal cancer could be used for targeted therapies for patients.
Yu G et al. [11]	2021	An SSL-based method on the mean teacher architecture using WSIs of colorectal cancer from 8803 subjects.	8803 patients	SSL	SSL achieved results comparable to that of SL, with massive annotations. SSL dramatically reduces the annotations, which has great potential to effectively build sophisticated pathological AI platforms in practice.
Afshar S et al. [12]	2019	An artificial neural network model was proposed in this work. Among the miRNAs retrieved from the Gene Expression Omnibus dataset, four miRNAs with the best miRNA score were selected by ANN units.	200 patients	ANN	The ANN model effectively distinguished between cancerous and non-cancerous samples with high accuracy. Additionally, upon evaluation, the ANN model demonstrated an area AUC of 1, indicating excellent predictive performance. Furthermore, the regression coefficient between the ANN’s output and the expected output was also 1. The confusion matrix revealed that all non-cancerous patients were correctly identified as normal, while cancerous patients were accurately classified as having cancer.
Zhang X et al. [13]	2019	The NIR spectral data from 104 paraffin-embedded CRC tissue samples consisting of an equal number of the BRAF V600E mutant and wild-type ones calibrated and validated the CP-ANN model.	312 tissue patient samples	CP-ANN	The CP-ANN model achieved a calibration classification accuracy of 98.0% and a validation classification accuracy of 94.4%. The model demonstrated a diagnostic sensitivity of 100.0% for the BRAF V600E mutation, a diagnostic specificity of 87.5%, and an overall diagnostic accuracy of 93.8%. Furthermore, it successfully distinguished between the BRAF V600E mutant and the wild type based on inherent differences, leveraging a dataset comprising 312 CRC tissue samples that were paraffin-embedded, deparaffinized, and stained.
Coppede F et al. [14]	2015	Promoter methylation was evaluated using methylation-sensitive, high-resolution melting and genotyping through the PCR-RFLP technique. The data underwent analysis using the Auto Contractive Map, a unique type of artificial neural network (ANN) capable of determining the strength of each variable’s association with all others. Additionally, it visually depicted the map of the primary connections within the data.	83 tissue patient samples	ANN	ANNs revealed the complexity of the interconnections among factors linked to DNA methylation in CRC.
Wallace MB et al. [15]	2022	Patients participating in colorectal cancer (CRC) screening or surveillance across eight centres (Italy, UK, US) were randomized into two groups. Each group underwent two consecutive colonoscopies on the same day, one with AI and one without AI. The order of colonoscopies with or without AI varied between the groups. The adenoma miss rate was calculated as the ratio of histologically confirmed lesions found during the second colonoscopy to the total number of lesions detected during both the first and second colonoscopies.	230 Patients	Gi-Genius	AI resulted in a two-fold reduction in miss rate of CRC neoplasia, supporting AI-benefit in reducing perceptual errors for small and subtle lesions at standard colonoscopy.
Glissen Brown JR et al. [16]	2022	A prospective, multi-center, single-blind randomized tandem colonoscopy investigation was conducted to assess a deep-learning-based Computer-Aided Detection (CADe) system. The study enrolled patients from four academic medical centres in the United States between 2019 and 2020. Participants undergoing CRC screening or surveillance were randomly assigned to either receive CADe colonoscopy or high-definition white light colonoscopy first. They underwent the other procedure immediately afterward, performed by the same endoscopist in tandem.	232 patients	Endoscreener	In this U.S., multicentre, tandem colonoscopy, randomized, controlled trial, a decrease in AMR and SSL misses rate, and an increase in first-pass APC with the use of a CADe-system, was observed when compared with HDWL colonoscopy alone.
Wang P et al. [17]	2019	In an open, non-blinded trial, consecutive patients were prospectively randomized to undergo diagnostic colonoscopy, either with or without the aid of a real-time automatic polyp detection system. This system provided simultaneous visual cues and sound alarms upon detecting polyps. The primary measured outcome was the adenoma detection rate.	1058 patients	SegNet	In a low-prevalence adenoma detection rate population, an automatic polyp detection system during colonoscopy resulted in major increases in the number of diminutive adenomas detected, as well as an increase in the rate of hyperplastic polyps.
Hamabe A et al. [23]	2022	MRI images were utilized as training data, and the resected specimen from 103 cases was processed into a circular shape. Ground-truth labels were created by annotating MR images with segmentation labels representing the tumor area, based on pathologically confirmed lesions. Furthermore, labels were assigned to the rectum and mesorectum areas. Subsequently, an automatic segmentation algorithm was developed, employing a U-net deep neural network.	201 patients	U-Net	This algorithm can provide an objective analysis of MR images at any institution, and aid in risk stratification in rectal cancer and the tailoring of individual treatments. Moreover, it can be used for surgical simulations.
Wu QY et al. [25]	2021	Data were collected from patients retrospectively as research objects. Faster R-CNNs were used to build the platform and the platform was evaluated according to the receiver operating characteristic curve.	183 patients	Faster R-CNN	Utilizing Faster R-CNN AI could potentially serve as an efficient and unbiased approach to establish a platform for predicting T-staging in rectal cancer.
Collins T et al. [26]	2021	A dataset comprising 12 patients with colon data and 10 patients with esophagogastric data was used to train various state-of-the-art machine learning techniques for cancer tissue detection through hyperspectral imaging. These methods include Support Vector Machines with radial basis function kernels, Multi-Layer Perceptrons, and 3D Convolutional Neural Networks (3DCNNs).	22 patients	3DCNN, SVM, MLPs and radial basis function kernels.	In this study, the 3DCNN model demonstrated better accuracy compared to classical machine learning models (MLP and RBF-SVM) in detecting esophagogastric and colon cancer. Despite the limited sample size, the findings show promise. While combining datasets could significantly enhance the performance of MLP and RBF-SVM models, the 3DCNN model did not benefit from this approach. This contradicts the common belief that CNNs necessitate larger datasets for training compared to other methods.
Muniz FB et al. [27]	2023	The proposed method consists of modelling hyperspectral data into a voxel format for pattern detection of each voxel using fully connected deep neural network.	55 patients	FCNN	The experiments utilized the K-fold cross-validation protocol with an interpatient approach, yielding an impressive overall accuracy of 99% with a deep neural network and 96% with a linear support vector machine. These results underscore the method’s exceptional ability to characterize tissues through deep learning and hyperspectral images.
Choi K et al. [30]	2020	By applying deep learning to develop a computer-aided diagnostic (CAD) system of colorectal adenoma, 3000 colonoscopic images were divided into 4 categories according to the final pathology: normal, low-grade dysplasia, high-grade dysplasia, and adenocarcinoma. Through the implemention of three convolutional neural networks using Inception-v3, ResNet-50, and DenseNet-161 as baseline models, the models were adjusted using several strategies: replacement of the top layer, transfer learning from pre-trained models, fine-tuning of the model weights, rebalancing and augmentation of the training data, and 10-fold cross-validation.	3000 colonoscopic patient images	CNN model using Inception-v3, ResNet-50 and DenseNet-161	In the experiments, the CNN-CAD system demonstrated the highest performance, achieving a classification accuracy rate of 92.48%. Across all criteria, the CNN-CAD results surpassed those of endoscopic experts. The model’s visualization outcomes revealed reasonable regions of interest, aiding in explaining pathology classification decisions. The study concluded that the CNN-CAD system effectively discerns colorectal adenoma pathology, outperforming the group of endoscopic experts.
